# Factors Influencing the Intention and Uptake of COVID-19 Vaccines on the African Continent: A Scoping Review

**DOI:** 10.3390/vaccines11040873

**Published:** 2023-04-20

**Authors:** Damian Naidoo, Anna Meyer-Weitz, Kaymarlin Govender

**Affiliations:** 1 Discipline of Psychology, School of Applied Human Sciences, Howard College, University of KwaZulu-Natal, Durban 4041, South Africa; 2 Health Promotion Unit, KwaZulu-Natal Department of Health, Pietermaritzburg, Private Bag X9051, Pietermaritzburg 3200, South Africa; 3 HEARD, College of Law and Management Studies, University of KwaZulu-Natal, Durban 4041, South Africa

**Keywords:** intention, barriers, uptake, facilitators, COVID-19 vaccines, Africa, scoping review

## Abstract

The COVID-19 pandemic is a severe concern worldwide, particularly in Africa. Vaccines are crucial in the fight against the COVID-19 pandemic. This scoping review examined existing literature from 2020 to 2022 on individual, interpersonal, and structural barriers and facilitators to COVID-19 vaccination within Africa to facilitate more informed health promotion interventions to improve vaccine uptake. This review was conducted using Arksey and O’Malley’s five-stage methodological framework. A comprehensive search was undertaken from 2021 to 2022 using six electronic databases: EBSCOhost, PubMed, Web of Science, ProQuest, WorldCat Discovery, and Google Scholar. Data was collected, charted into themes, and summarized using a standard data extraction sheet in Microsoft Excel. A total of forty (*n* = 40) published academic articles were reviewed, with many conducted in Nigeria (*n* = 10), followed by Ethiopia (*n* = 5) and Ghana (*n* = 4) and the rest elsewhere in Africa. Thematic narratives were used to report data into six themes: attitudes and perceptions about COVID-19 vaccines, intention to uptake COVID-19 vaccines; factors and barriers associated with COVID-19 vaccine uptake; socio-demographic determinants affecting the intention and uptake; and information sources for COVID-19 vaccines. The intention for uptake ranged from 25% to 80.9%, resulting in a suboptimal uptake intention rate (54.2%) on the African continent. Factors that promoted vaccine acceptance included confidence in the COVID-19 vaccines and the desire to protect people. Age, education, and gender were the most common factors significantly associated with vaccine acceptance. Most studies revealed that considerable barriers to vaccine uptake exist in Africa. Concerns about potential side effects, vaccine ineffectiveness, a perceived lack of information, and inaccessibility were among the individual, interpersonal, and structural barriers to COVID-19 vaccine uptake. The unwillingness to receive the COVID-19 vaccine was strongly correlated with being female. Mass and social media were the main sources of information regarding COVID-19 vaccines. To encourage vaccine uptake, governments should pay attention to refuting misinformation through integrated community-based approaches, such as creating messages that convey more than just information.

## 1. Introduction

The novel coronavirus 2019, termed COVID-19, is a highly transmissible and pathogenic viral infection caused by severe acute respiratory syndrome coronavirus 2 (SARS-CoV-2) [1,2,3], and COVID-19 vaccines are seen as an effective public health tool in mitigating the spread of SARS-CoV-2. The COVID-19 pandemic has raised many direct and indirect health problems [3]. Although most people experience upper respiratory tract and pulmonary symptoms, those with severe COVID-19 may also experience widespread small and large vessel thrombosis, microvascular injury, cardiac conduction abnormalities, neurologic deficits, diarrheal symptoms, gastrointestinal bleeding, organ dysfunction, hypercytokinemia, and lymphopenia, any of which can be life-threatening [4,5]. More than three years since the first SARS-CoV-2 was first identified in Wuhan, China [6,7], the development of COVID-19 vaccines has accelerated at an unprecedented rate [8], and the WHO has approved several vaccines against COVID-19 that have been distributed globally in various regions at different stages [9]. 

SARS-CoV-2 has spread to most African countries [10], and the continent has, to date, received around 500 million doses of the COVID-19 vaccine and administered 327 million doses [11]. As of January 2022, Africa accounted for approximately 3.3% of global cases and 4.2% of global deaths. However, it is of concern that only 10% of Africa’s population is fully vaccinated [12].

Vaccine uptake is dependent on various psychological states, behaviors, and contextual factors. More specifically, vaccine hesitancy (VH) refers to indecisiveness or uncertainty among individuals about vaccine uptake, and those vaccine-hesitant individuals can exhibit a spectrum of attitudes [13], dependent on individual and environmental influences. However, vaccination refusal is classified as a decision to reject vaccination [14]. The current literature has noted a number of barriers related to vaccine uptake, i.e., inadequate infrastructure, prevailing cultural norms, poor health literacy, vaccine-related myths, conspiracy theories, and misinformation [15,16,17]. 

Despite recent systematic reviews in low- and middle-income countries (LMICs) [18,19,20], this review aims to synthesize and map the current literature specifically in African countries, which are not well represented in the above reviews due to the persistent challenges in the rollout of vaccines and subsequent vaccination of the population on the African continent. We furthermore intend to extend the focus on COVID-19 vaccine uptake and VH to include more detailed information on the individual, interpersonal, and structural barriers and facilitators to COVID-19 vaccination.

Accordingly, this review attempts to explore factors influencing the intention and uptake of COVID-19 vaccines on the African continent. The present study will shed light on the existing literature by understanding the determinants of uptake and factors influencing people’s decisions to vaccinate against COVID-19, which is necessary to inform health promotion interventions to improve the uptake of COVID-19 vaccination in this context. The review addressed the following questions: (i) What are the perceptions and attitudes regarding COVID-19 vaccines among people in Africa? (ii) What are the facilitating factors associated with COVID-19 vaccine uptake in this geographical setting? (iii) What barriers are associated with COVID-19 vaccine uptake?

## 2. Materials and Methods

This study utilized a scoping review approach because of its ability to identify trends and gaps in an existing knowledge base to inform research, policy, and practice [21]. This scoping review was conducted following the analytic framework of Arksey and O’Malley [22]. Arksey and O’Malley developed a five-stage methodological framework to guide researchers in conducting scoping reviews [23]. 

The following five-stage framework proposed was as follows: (1) identifying the research questions; (2) searching for relevant studies; (3) selecting studies; (4) charting the data; and (5) collating, summarizing, and reporting the results [22] (p. 22). A review protocol for ethical clearance was submitted to the University of KwaZulu-Natal (UKZN) Humanities and Social Sciences Research Ethics Committee (HSSREC).

### 2.1. Searching for Relevant Studies

A systematic search was conducted utilizing the relevant electronic databases such as EBSCOhost, PubMed, Web of Science, ProQuest, WorldCat Discovery, and Google Scholar. The search followed a process to identify studies that addressed the research questions outlined above. These four electronic databases (EBSCOhost, PubMed, Web of Science, and Google Scholar) were searched from 1 October 2021 to 13 October 2021. Another search was conducted via the remaining two electronic databases (ProQuest and WorldCat Discovery) from 25 October 2022 to 8 November 2022. The following restrictions were placed on all four databases to produce the relevant studies needed for this scoping review. Studies were searched from 2020 to 2022 and published in English and in peer-reviewed journals.

The COVID-19 pandemic was the motivating factor behind this timeline. Additional limiters were placed to only search for full-text studies conducted on the African continent. The following search terms were included: “COVID-19 vaccines”, “COVID-19”, “SARS-CoV-2 vaccines”, “perceptions”, “attitudes”, “barriers”, “drivers”, “acceptance”, “hesitancy”, “Africa”, “vaccine uptake”, “vaccine refusal”, “COVID-19 vaccination uptake”, “COVID-19 vaccination intention”, and “COVID-19 vaccine willingness”. The final search strategies for EBSCO host can be found in the Appendix A Table A1.

### 2.2. Study Selection

After conducting a complete title and abstract screening in the databases mentioned above, studies were screened using the Population–Concept–Context (PCC) framework to establish their eligibility for this review. The researcher excluded all studies that did not answer the review’s research query: published research on psychosocial and contextual factors influencing intention and uptake of COVID-19 vaccines in Africa.

Furthermore, the inclusion and exclusion criteria guided the selection of full-text studies to determine which studies were most appropriate to include in this review. The researcher focused on quantitative, qualitative, and mixed-methods academic/published journals (peer-reviewed journals) published in English between 2020 and 2022 on attitudes, beliefs, barriers, factors, facilitators, perceptions, acceptance, intentions, concerns, uptake, and hesitancy toward COVID-19 vaccines on the African continent among the general population. The researcher excluded gray literature (i.e., unpublished journals, reports, documents, conference papers, memoranda, theses, letters, and protocols), summaries, and other reviews. Additional exclusions included studies that focused solely on populations other than the general population (e.g., HCWs, high-risk populations, university students, and academics).

### 2.3. Charting Data

A standardized data extraction sheet in Microsoft Excel was used to collate and chart the data into themes and summarize studies and reports. The following headings were used to extract detailed information for the included studies: authors and year of publication; study setting, i.e., country and data collection period; and methodology. The methodology section consisted of study characteristics, i.e., study design, population target, and sample size.

Due to the heterogeneity of studies, a narrative synthesis approach was used to collect, synthesize, and map the literature [23]. The following categories were used to categorize the studies: (1) attitudes and perceptions towards COVID-19 vaccines; (2) intention to take the COVID-19 vaccines; (3) reasons for acceptance or non-acceptance; (4) determinants affecting the vaccine-related outcome; and (5) information sources for COVID-19 vaccines. The researcher applied thematic narratives to report all data [24].

## 3. Reporting the Results

**Figure 1:**
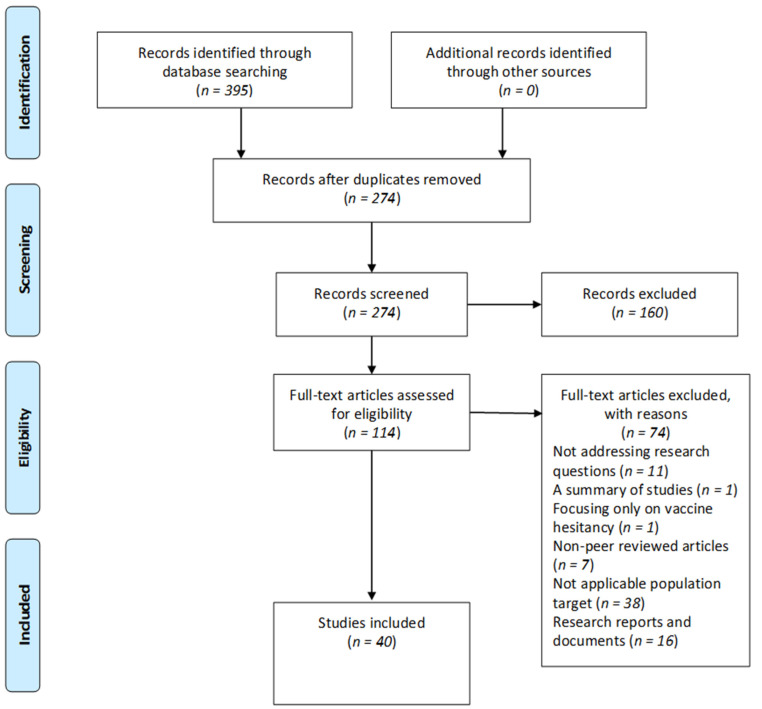
PRISMA flow diagram: selection of included studies.

In the initial search, 395 studies were identified from database searches: EBSCOhost (*n* = 247), PubMed (*n* = 53), Web of Science (*n* = 43), ProQuest (*n* = 26), WorldCat Discovery (*n* = 13), and Google Scholar (*n* = 13). After removing duplicates with EndNote (V.X9), 274 studies were screened by title and abstract to find those that met the initial screening criteria. One hundred and sixty (*n* = 160) studies were excluded because they were irrelevant to the research question, leaving 114 studies for full-text review. Following the inclusion and exclusion assessment criteria, studies were further excluded because they did not address research questions (*n* = 11), a summary of studies (*n* = 1), focusing only on VH (*n* = 1), non-peer-reviewed articles (*n* = 7), not applicable population targets (*n* = 38), and research reports and documents (*n* = 16), resulting in 40 published articles for the final analysis. The PRISMA flow diagram below illustrates the selection process in Figure 1.

### 3.1. Study Design of Included Studies

Many studies (*n* = 37) that were conducted used a quantitative research approach. Of those studies, (*n* = 33) adopted a cross-sectional design. Two studies (*n* = 2) adopted a qualitative research approach, and one (*n* = 1) applied a mixed-methods research approach.

### 3.2. Country of Focus

See Table 1. For the number of countries included in this review. The multiple studies reviewed were from Bono et al. [25], who examined nine LMICs. Bono et al. sought to examine the factors influencing the COVID-19 vaccine’s acceptability in various LMICs across three continents. However, only five African countries surveyed were considered, including the DRC, Benin, Uganda, Malawi, and Mali. Ahiakpa et al. [26] surveyed 18 countries: Nigeria, Somalia, Ghana, Mozambique, Kenya, Rwanda, Tanzania, Uganda, Zambia, Ethiopia, South Africa, Malawi, Morocco, Botswana, Cameroon, the DRC, Eswatini (formerly Swaziland), and Djibouti. The aim of Ahiakpa et al.’s review sought to assess COVID-19 vaccine uptake among adult Africans. In addition, Anjorin et al. [27] conducted a multinational study to assess potential VH on the African continent. They surveyed 13 countries, including Liberia, South Africa, Malawi, Sudan, Tanzania, Morocco, Nigeria, Egypt, Rwanda, Ghana, Kenya, the DRC, and Cameroon. The current review provides a nuanced approach compared to other reviews, which goes beyond identifying COVID-19 vaccine acceptance and VH. This review explores various drivers and barriers affecting COVID-19 vaccine acceptance, intention, and hesitancy among the general population on the African continent rather than focusing on Sub-Saharan Africa.

### 3.3. Participant Characteristics 

The general adult population (aged ≥ 18 years) (*n* = 30) made up most of the study sample, while the general population comprised (*n* = 10). Three studies by Elhadi et al. [28]; Gunawardhana et al. [29]; and Toure et al. [30] focused on the general population, including HCWs (*n* = 2) and pregnant women (*n* = 1). However, these sub-populations were not considered due to the abovementioned exclusion criteria. Only the general population sample was used. The sample sizes for the current review ranged from 14 to 11,971 respondents. Male respondents ranged from 15% to 91.7%, while female respondents ranged from 8.3% to 100%. Additional baseline characteristics of the selected studies are listed in Table 2.

## 4. Results Pertaining to the Research Questions

The findings of the six themes are discussed in this section. See Table 3. for the predictors of COVID-19 vaccine uptake among the general population in Africa.

### 4.1. Attitudes and Perceptions Regarding COVID-19 Vaccines among People in Africa

In this review, a total of eighteen studies (*n* = 18) reported on attitudes or perceptions regarding COVID-19 vaccines in Africa. Six of those studies (*n* = 6) found that the participants were hesitant toward the vaccine [29,32,37,39,44,60]. Furthermore, of the 18 studies, three studies each reported a positive [28,48,53] or a negative [31,55,59] attitude toward the vaccine. While the qualitative study by Chauke et al. [38] reported opposing attitudes among respondents regarding the COVID-19 vaccines.

Two of the 18 studies reported a positive perception [34,46] of the vaccine, while five of the 18 studies reported an overall negative perception [26,30,48,53,56] of the vaccine. According to the findings, two studies were diametrically opposed regarding attitudes and perceptions toward the vaccine. The study among the general adult populations by James et al. [48] in Nigeria and Mesesle [53] in Ethiopia discovered an overall positive attitude toward the vaccine. However, respondents also expressed a negative perception of the COVID-19 vaccine. The relationship between attitude and behavior is not always consistent [65,66], with behaviors being influenced by attitudes and various other factors impacting one’s decision or willingness to uptake vaccines [67,68]. 

### 4.2. Intention to Uptake COVID-19 Vaccines

**Figure 2:**
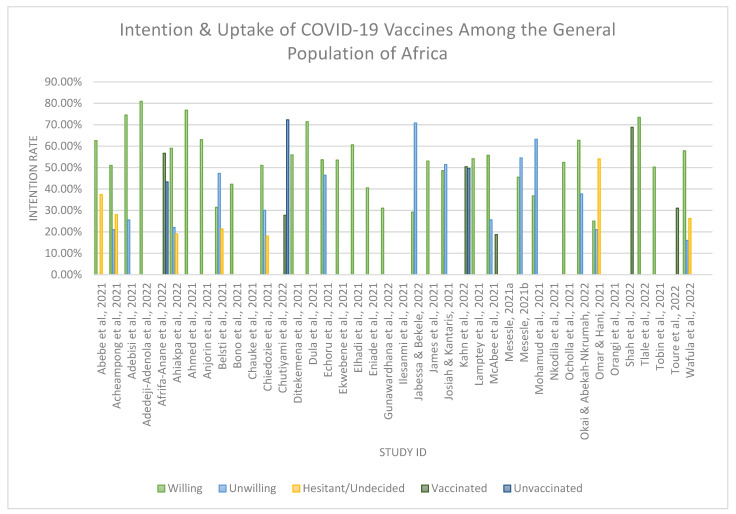
An illustration of COVID-19 vaccine uptake rates among the included studies in Africa. [25,26,27,28,29,30,31,32,33,34,35,36,37,39,40,41,42,43,44,45,47,48,49,50,51,52,54,55,57,58,59,61,62,63,64].

Thirty-five (*n* = 35) studies assessed the willingness, refusal, hesitancy, vaccinated, and unvaccinated rates regarding COVID-19 vaccination among the general African population. (see Figure 2).

Thirty (*n* = 30) studies [25,26,27,28,29,31,32,33,34,36,37,39,41,42,43,44,45,47,48,49,51,52,54,55,57,58,59,62,63,64] examined respondents’ intention to uptake the COVID-19 vaccine, while the remaining five studies did not mention the intention rate however reported on participants being vaccinated against COVID-19.

Over the thirty (*n* = 30) studies, the intention for uptake ranged from 25% to 80.9%. Only nine studies (*n* = 9) reported a lower-than-average rate (i.e., below 50%). In comparison, twenty-one studies (*n* = 21) illustrate an intention rate of 50% or higher (see Table 2. for a detailed intention rate). The country with the lowest intention rate was reported as Egypt (25%), by Omar and Hani [59]. In contrast, the country with the highest intention rate was reported as Nigeria (80.9%) by Adedeji-Adenola et al. [34]. The average intention rate to uptake the COVID-19 vaccines among the included studies was 54.2%, resulting in a suboptimal uptake rate on the African continent.

The qualitative study conducted in South Africa by Chauke et al. [38] among 14 youth participants (aged 18–35 years) did not statistically report the intention to accept the COVID-19 vaccine but revealed mixed feelings among the young population. Some young people considered it necessary to take the vaccine to mitigate the effects of the pandemic. Others, on the other hand, believe that the vaccine should only be used as a last resort because it negatively affects their genetic makeup, including their reproductive system. Furthermore, these participants believed that, once vaccinated, their daily activities would be monitored through a microchip in the COVID-19 vaccine.

According to the study by Bono et al. [25], the average intention rate was 42.2%, with wide ranges between 22.6% and 65.4%. Uganda was reported to have the highest intention for uptake among the five African countries. On the other hand, Benin was found to have the lowest intention for uptake. Ahiakpa et al. [26] reported on 17 African countries with an average intention rate of 59% to uptake the COVID-19 vaccine, while 22% of participants refused to take the COVID-19 vaccine regardless of the directive given by their governments, and 19% were undecided on taking the vaccine. The continent-wide cross-sectional study by Anjorin et al. [27] assessed the willingness to accept the COVID-19 vaccine. The average acceptance rate was 63%, and they agreed to accept the vaccine as soon as possible. Liberia reported the highest intention rate of 84%, while the lowest intention rate of 33% was reported in Cameroon.

Jabessa and Bekele [47] reported the highest rejection rate (70.8% in Ethiopia). Omar and Hani [59] reported the highest VH rate (54% in Egypt) for uptake of the COVID-19 vaccine. The subsequent studies reported that participants had been vaccinated against COVID-19 [30,35,40,50,52,61]. Of those three studies [35,50,61], it was indicated that above 50% of their participants were vaccinated with at least one dose. Shah et al. [61] reported the highest vaccination rate (68.8% in Kenya) in this review.

### 4.3. Factors Associated with COVID-19 Vaccine Uptake

Various factors that promoted the intention for vaccine uptake were reported in thirteen studies (*n* = 13). Across the 13 studies these were grouped as confidence in the COVID-19 vaccine [28,32,39,42,44] and the desire to protect others, e.g., family, community members, and vulnerable people [32,35,38,63]. A less common reason was that the acceptance of the vaccine is a public responsibility [32]. In addition, other reasons relate to being better informed about COVID-19 i.e., having an awareness of the possible side effects of the COVID-19 vaccine [32]; increased COVID-19 vaccine education [35]. Further reasons were observing others receive the COVID-19 vaccine; having free access to the COVID-19 vaccine [35]; receiving a vaccine certificate [35]; prior diagnosis of COVID-19 [26]; having a positive perception of the COVID-19 vaccine [26]; returning to normalcy by opening up the economy [38]; COVID-19 vaccine from an African country [29,49]; COVID-19 vaccine from a Muslim country [49]; having access to media [54]; having a high perceived susceptibility of contracting COVID [60]; the presence of comorbidities [63]; COVID-19 vaccines being recommended by HCWs and for self-protection [63].

According to findings from Afrifa-Anane et al. [35], a qualitative study in Ghana among women revealed two themes i.e., interpersonal and structural factors, that promoted vaccine acceptance. The desire to protect oneself and one’s family is to get vaccinated against COVID-19, and seeing others get the COVID-19 vaccine was the main interpersonal factor that facilitated the uptake of the COVID-19 vaccine among respondents. While the structural facilitators included being educated about COVID-19 vaccines and the vaccine being free of charge, receiving a vaccination certificate and giving souvenirs to vaccinated people facilitated COVID-19 vaccine uptake. In this instance, a vaccination certificate or passport is required to access social services (such as banks), employment, and international travel.

### 4.4. Barriers to the Uptake of the COVID-19 Vaccine

Thirty-three studies (*n* = 33) highlighted the barriers associated with COVID-19 vaccine uptake. Of those twenty-nine studies (*n* = 29) cited fears over potential side effects and the newly developed vaccine being unsafe for the African population [27,29,30,32,33,34,35,36,37,40,41,42,43,44,45,48,50,54,55,56,57,58,59,60,61,62,63,64]. Eleven studies (*n* = 11) reported concerns regarding the vaccine’s ineffectiveness in protecting against COVID-19 [29,32,36,40,41,42,55,56,60,63,64], resulting in a general apprehension about being vaccinated. Perceptions based on conspiracies about the COVID-19 vaccine were also cited in eight studies (*n* = 8) (e.g., the vaccine was designed to kill people in Africa, the vaccine was designed to sterilize the African population, and the vaccine causes COVID-19) [26,28,29,35,39,41,42,56]. Five studies each (*n* = 5) attributed barriers to a perceived lack of information to make informed decisions about its uptake [29,32,50,54,64] and scientific uncertainty [33,49,55,58,62] (e.g., mistrust in science or the vaccine, the vaccine has not gone through enough clinical trials, or the quality of COVID-19 vaccines sent from Western countries was not effective against COVID-19 in Africa). Three studies each (*n* = 3) reported on vaccine inaccessibility [32,35,50]—demanding work schedules, vaccine shortages, long queues, and hard-to-access vaccination sites, a lack of trust in stakeholders [30,38,45] (e.g., vaccine manufacturers and the government), who believed that their immune systems would protect them from contracting COVID-19 [33,36,64]; participants doubted the seriousness of the pandemic [36,49,63]. A low perceived risk of disease was reported in two studies (*n* = 2) [58,64]. Respondents also had religious beliefs (e.g., the vaccine contains the mark of the beast) [44,60], and would not accept the COVID-19 vaccine from Western or European countries [29,49], which resulted in poor vaccine uptake. One study (*n* = 1) reported on participants being afraid of needles [63], having a negative perception of the vaccine [30], having a prior diagnosis of COVID-19 [64], and preferring alternative treatments to the COVID-19 vaccine (e.g., drugs such as hydroxychloroquine, azithromycin, and ivermectin) [27] and negative past experiences with vaccines [40].

Individual, interpersonal, and structural barriers to COVID-19 vaccine uptake were identified [35] among Ghanaian women. Individual barriers to COVID-19 vaccine uptake included tight work schedules, vaccine effectiveness, and being pregnant. Subscribing to misinformation or conspiracy theories about COVID-19 emerged as an interpersonal barrier to COVID-19 vaccine uptake. Vaccine-related misconceptions include the idea that being injected with COVID-19 vaccines will affect one’s reproductive system, such as causing barrenness in women and impotence in men. Furthermore, vaccines are made to make people foolish and are intended to kill the African population. The structural barriers identified were long queues at vaccination centers, accompanied by vaccine shortages and proximity to a vaccination center.

### 4.5. Socio-Demographic Determinants Affecting the Intention and Uptake of COVID-19 Vaccines

Twenty-seven studies (*n* = 27) reported socio-demographic determinants influencing COVID-19 vaccine intention and uptake. Twenty studies (*n* = 20) found a statistically positive relationship between socio-demographic characteristics and vaccine uptake. Age [28,30,31,32,33,37,43,45,47,48,50,62,63,64], educational level [29,30,31,34,37,43,47,48,49,52,54,62], and gender [32,37,48,49,52,54,62,63] were shown to be significant predictors of vaccination uptake. Furthermore, ten studies [28,30,31,32,33,37,45,47,48,50,62,64] indicated that older participants (30 ≥ years) were more likely to accept the vaccine than their counterparts. In five studies (*n* = 5) marital status [28,30,37,43,48] was significantly associated with vaccine uptake. Four studies (*n* = 4) suggested an income level [41,43,47,49] and religious affiliation [37,43,49,63], while in three studies (*n* = 3) occupation [37,43,57] was a predictor of vaccine uptake.

Furthermore, three studies indicated other socio-demographic determinants, such as geographic region [28,33,64], prior diagnosis of COVID [28,34,41], having a high-risk perception for contracting COVID-19 [45,49,64], employment status [47,49,62], and confidence in the COVID-19 vaccine [30,50,52]. While two studies each indicated having a chronic illness [31,62], trust in stakeholders, e.g., vaccine developers [49,63], and adopting good preventive practices towards COVID-19, e.g., getting vaccinated [52], handwashing [52], and willingness to wear a mask [62], were significantly associated with COVID-19 vaccine uptake. Other significant predictors of vaccine acceptance reported by one study each were having adequate knowledge of COVID-19 [31]; residence [43]; vaccine efficacy of 70% [28]; being a parent [29]; knowing someone who tested positive for COVID [28]; COVID-19 vaccines being recommended by HCWs [52]; COVID-19 vaccine provided free of charge [52]; vaccine accessibility [52]; the use of mass media [54]; received childhood vaccines [54]; willing to pay and travel for being vaccinated against COVID-19 [63]; receiving a vaccine during the outbreak [30]; not being pregnant [30]; vaccine eligibility [30] and developing severe COVID and side effects from COVID-19 [64].

According to Jabessa and Bekele [47], Ethiopian rural residents reported a statistically positive relationship between socio-demographic characteristics and vaccine uptake, which is inconsistent with the other studies (*n* = 19) included in this review. Having a low perception of the use of vaccination; being unemployed, having a low level of acceptance of COVID-19 vaccines, being unwilling to test for COVID, and having an extremely low (illiterate) education level were significant predictors for uptake.

Further analysis indicated that nine studies (*n* = 9) revealed a statistically negative relationship between socio-demographic characteristics and vaccine uptake, resulting in the rejection of the COVID-19 vaccine. Gender [27,36,59] and residence [27,59,60] were significant in not accepting the COVID-19 vaccine. Female respondents were cited in all three studies, and residing in an urban area [27,59] was associated with an unwillingness to vaccinate. Three studies each indicated non-acceptance of the COVID-19 vaccine based on education level [33,37,59]. Two studies each cited barriers such as having a negative experience with vaccines [27,40], marital status [37,58], age [27,33], safety concerns [40,60], and effectiveness concerns [40,60]. One study reported on: Low employment status [27], low income level [27], low occupation [37], scientific uncertainty [40], loss of someone to COVID-19 [28], rejection of the flu vaccine [59]; lack of trust in stakeholders; [59]; vaccine inaccessibility [60], higher vaccine knowledge [30], and having a negative attitude towards COVID-19 vaccines [30].

### 4.6. Information Sources for COVID-19 Vaccines

**Figure 3:**
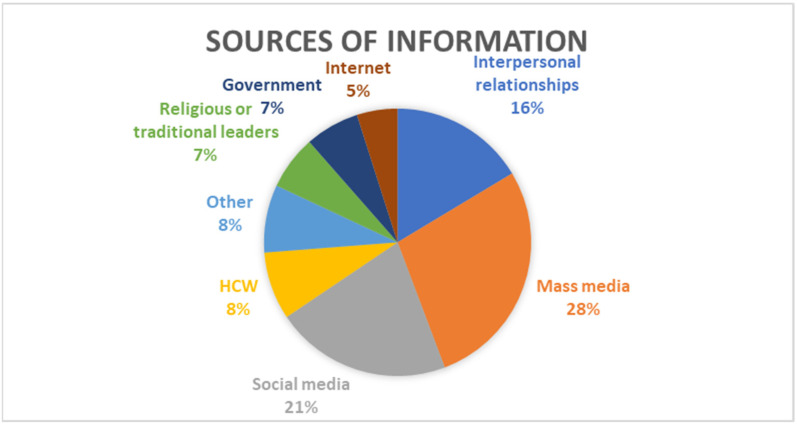
Information sources regarding COVID-19 vaccines.

Seventeen studies (*n* = 17) reported on information sources [26,27,28,29,30,31,32,34,41,45,53,54,57,62,63,64] regarding COVID-19 vaccines (See Figure 3). below illustrates the information sources regarding COVID-19 vaccines in the included studies. The most common source of information about COVID-19 vaccines was mass media (*n* = 17) [26,27,28,29,30,31,32,34,41,45,53,54,57,62,63,64], which included radio, TV, newspapers, and magazines, followed by social media (*n* = 13) [26,28,29,31,32,34,41,45,46,57,62,63,64] and interpersonal relationships (*n* = 10) [26,29,30,31,32,33,34,41,57,63], which included information from family, friends, neighbors, and colleagues. Five studies (*n* = 5) reported respondents receiving information from HCWs [27,29,34,57,63] and from other sources [26,27,29,32,34], which include scientists, celebrities, medical aid, and academic journals.

Four studies (*n* = 4) reported on participants who received their information from religious and traditional leaders [26,27,29,41], from governments and governmental officials, i.e., politicians [27,29,30,62] and participants receiving their information from the internet [28,31,62,63], which included sites such as the Centers for Disease Control and Prevention (CDC), the Nigeria Centre for Disease Control (NCDC), and the World Health Organization (WHO).

## 5. Discussion

The success of Africa’s extensive COVID-19 pandemic vaccination program [69] depends on high vaccination rates. As a result, vaccination uptake and acceptance are critical in the fight against COVID-19 [70]. To increase vaccine acceptance, it is necessary to understand the factors that influence vaccine intention and uptake of COVID-19 vaccines in order to inform interventions in this regard.

Forty published academic journal articles were reviewed to gain a more in-depth and nuanced understanding of how various factors, such as psychosocial and contextual factors, influence COVID-19 vaccine uptake intentions and behaviors among people in Africa. Most of the studies included in this review were quantitative cross-sectional studies conducted in Nigeria. The findings of this review revealed a varied response in people’s perceptions and attitudes regarding COVID-19 vaccines. There was a general hesitancy regarding the uptake of COVID-19 vaccines; only 54.2% of studies reported a higher-than-average intention to uptake the COVID-19 vaccines. 

The most frequently cited demographic factors influencing COVID-19 determinants of vaccine intention and uptake in this review were the respondents’ age, education, and gender. Men and older adults aged 30 years and older are more likely to accept the vaccine, similar to a study conducted in Slovenia by Petravi et al. [71], who reported that being male and middle-aged was associated with better vaccine uptake. This may stem from beliefs about being at higher risk of contracting COVID-19 and the higher severity of the illness. Therefore, vaccination is likely to be accepted by those who perceive themselves as being at a higher risk of contracting COVID-19 [46,72,73].

The low intention rate is due to the rapid development of COVID-19 vaccines, concerns about the vaccines’ safety and effectiveness, and mainly reports on the adverse side effects [37,72,74]. This is exacerbated by misinformation, which has fostered distrust in government officials, regulatory agencies, and pharmaceutical companies [25,74,75]. The fact that social media was reported as a source of information regarding COVID-19 and vaccines explains the role of misinformation and conspiracy theories in VH. Furthermore, the dissonance between holding opposing views, i.e., messages from significant others i.e., government and HCW, as well as social media, is likely to create barriers to the uptake of preventative measures [76]. Health communication messages should therefore be directed toward countering fake news regarding COVID-19 to enhance acceptance of COVID-19 vaccines. 

Numerous significant barriers to COVID-19 vaccine uptake in Africa have been identified, particularly around distrust in vaccines, safety concerns, and vaccine effectiveness [77]. A history of medical experimentation has caused significant mistrust of Western medicine in Africa [78]. The mistrust of Western medicine fuelled by socio-political issues, which are founded on historical and contemporary racism, has also eroded vaccine trust [79,80]. The findings by Josiah and Kantaris [50] in Nigeria and Gunawardhana et al. [29] in Cameroon showed acceptance of COVID-19 vaccines when obtained from an African country rather than Western or European countries. This seems to be a clear call for African countries to play a more active role in vaccine development and distribution [81]. In a context of distrust, negative experiences with vaccine safety are likely to impact uptake and further raise questions about vaccine effectiveness. In the early 2000s, HIV vaccine trials were abruptly halted in South Africa due to many recipients developing increased susceptibility to infectious diseases [78]. Trust in science and scientists is strongly correlated with vaccine confidence [82]. Therefore, confidence in vaccines is expressed as trust in individual vaccines and/or trust in the health care systems [83] and trust in the government [84]. The ability to comprehend and believe in the safety and effectiveness of vaccinations is a critical predictor of intention and vaccination uptake [85] and is important to counteract widespread misinformation [84].

Mohamud et al. [55] in Somalia reported that 73.8% of participants refused to vaccinate their children against COVID-19 once they became eligible for immunization. Religious and traditional leaders impact the general population’s intention to vaccinate, according to a study by Afrifa-Anane et al. [35] among women in Ghana. Respondents mentioned that some pastors advised their congregations not to accept the COVID-19 vaccine because it is demonic. Rather than taking these vaccines, pastors provided them with spiritual guidance to help protect them from COVID-19. The study conducted by Elhadi et al. [28] in Libya found that the death of a loved one from COVID-19 significantly decreased the likelihood of COVID-19 vaccine uptake. According to Jabessa and Bekele [47], findings revealed that a low education level, a low level of perception about the usefulness of vaccine, a low-income category, being unemployed, an older age, and an unwillingness to test for COVID-19 were predictors of willingness to receive the COVID-19 vaccine among residents of southwestern Ethiopia. Further qualitative studies are required to explore this phenomenon. Attitudes and behaviors are not always completely aligned. An individual’s behavior can be influenced by a combination of beliefs, perceptions, environmental needs, and self-preservation [86].

Most studies emphasize the importance of stakeholders educating and raising the level of awareness among the general public about COVID-19 vaccines with consideration of cultural orientations e.g., collectivism, to foster social responsibility for COVID-19 prevention, including vaccination prosociality, which has been found to be a significant positive predictor of COVID-19 vaccination intention [87]. Therefore, efforts are necessary to combat the effects of misinformation by providing easily accessible information to the general public through multiple platforms, including mainstream and social media. 

### Strengths and Limitations

Although this study adopted an inclusive approach, many articles were quantitative and cross-sectional, which means that further in-depth qualitative research is needed to better understand the factors influencing the intention and uptake of COVID-19 vaccines on the African continent. Only peer-reviewed studies published in English were considered for this review.

## 6. Conclusions

As COVID-19 becomes endemic in many African countries, the uptake of COVID-19 vaccines is one key way to achieve immunity and mitigate the negative impact of COVID-19. Vaccines are the most effective prevention method against severe COVID-19 complications and hospitalization. Most of the studies reviewed reported significant barriers to COVID-19 vaccine uptake, resulting in suboptimal intention rates. It is also noted that social media has exacerbated the effects of misinformation and conspiracy theories, resulting in divided communities where some support and others oppose COVID-19 vaccines.

Therefore, improving general health literacy and knowledge regarding COVID-19 vaccines among African populations is critical. It is now up to various stakeholders and policymakers to take effective action to provide tailored health promotion interventions with consideration of the personal, social, and contextual factors influencing vaccine acceptance and thus address the pandemic’s adverse health and socio-economic consequences.

This paper calls on the relevant stakeholders to train and create opportunities for Community Healthcare Workers (CHWs) and Health Promotion Practitioners (HPPs) to engage with the public through information, education, and communication platforms to improve vaccine literacy in general and for COVID-19 vaccines, as well as cultivate positive beliefs and attitudes towards COVID-19 vaccines. Greater awareness of social responsibility in protecting oneself, loved ones, and the vulnerable in communities may augur well for increased uptake of vaccines. However, COVID-19 vaccination uptake in the African context will hinge greatly on building trust between the general population, scientists, the health care system, and governments.

## Figures and Tables

**Table 1:** Illustrates—the number of countries reviewed.

Country of Focus	Number of Studies
Ethiopia	5
Ghana	4
Nigeria	10
Multiple African Countries	3
Somalia	2
South Africa	2
DRC	2
Mozambique	1
Uganda	2
Libya	1
Cameroon	1
Zimbabwe	1
Kenya	3
Egypt	1
Botswana	1
Guinea	1

**Table 2:** Characteristics and vaccine acceptance and intention rates.

Author(s) and Publication Year	Country and Data Collection Period	Methodology	Willing	Refusal	Hesitant/Undecided	Vaccinated	Unvaccinated
**Abebe et al., 2021** **[31]**	Ethiopia March 2021	**Study design:** A quantitative cross-sectional study **Population target:** General adult population (aged ≥ 18 years) **Sample size:** 492	62.6%		37.4%		
**Acheampong et al., 2021** **[32]**	Ghana February 2021	**Study design:** A quantitative cross-sectional study **Population target:** General population **Sample size:** 2345	51%	21%	28%		
**Adebisi et al., 2021** **[33]**	Nigeria August 2020	**Study design:** A quantitative cross-sectional study **Population target:** General population **Sample size:** 517	74.5%	25.5%			
**Adedeji-Adenola et al., 2022** **[34]**	Nigeria April to June 2021	**Study design:** A quantitative cross-sectional study **Population target:** General adult population (aged ≥ 18 years) **Sample size:** 1058	80.9%				
**Afrifa-Anane et al., 2022** **[35]**	Ghana October to November 2021	**Study design:** A cross-sectional descriptive qualitative design **Population target:** General Population–Women **Sample size:** 30				56.7%	43.3%
**Ahiakpa et al., 2022** **[26]**	17 African countries December 2020 to March 2021	**Study design:** A quantitative cross-sectional study **Population target:** General adult population (aged ≥ 18 years) **Sample size**: 365	59%	22%	19%		
**Ahmed et al., 2021** **[36]**	Somalia December 2020 to January 2021	**Study design:** A quantitative cross-sectional study **Population target:** General adult population (aged ≥ 18 years) **Sample size:** 4543	76.8%				
**Anjorin et al., 2021** **[27]**	Multiple African countries February to March 2021	**Study design:** A quantitative cross-sectional study **Population target:** General adult population (aged ≥ 18 years) **Sample size**: 5416	63%				
**Belsti et al., 2021** **[37]**	Ethiopia February to March 2021	**Study design:** A quantitative study **Population target:** General adult population (aged ≥ 18 years) **Sample size**: 1184	31.4%	47.3%	21.3%		
**Bono et al., 2021** **[25]**	Multiple African countries December 2020 to February 2021	**Study design:** A quantitative cross-sectional study **Population target:** General adult population (aged ≥ 18 years) **Sample size**: 621	42.2%				
**Chauke et al., 2021** **[38]**	South Africa	**Study design:** A qualitative study **Population target:** General population-Youth (18–35 years) **Sample size:** 14					
**Chiedozie et al., 2021** **[39]**	Nigeria	**Study design:** A quantitative study **Population target:** General adult population (aged ≥ 18 years) **Sample size:** 499	51%	30%	18%		
**Chutiyami et al., 2022** **[40]**	Nigeria October to December 2021	**Study design:** A quantitative population-based study **Population target:** General population **Sample size:** 577				27.7%	72.3%
**Ditekemena et al., 2021** **[41]**	The Democratic Republic of Congo August 2020 to September 2020	**Study design:** A quantitative cross-sectional study **Population target:** General population **Sample size:** 4160	55.9%				
**Dula et al., 2021** **[42]**	Mozambique March 2021	**Study design:** A quantitative cross-sectional study **Population target:** General adult population (aged ≥ 18 years) **Sample size:** 1878	71.4%				
**Echoru et al., 2021** **[43]**	Uganda July to September 2020	**Study design:** A quantitative cross-sectional study **Population target:** General adult population (aged ≥ 18 years) **Sample size:** 1067	53.6%	46.4%			
**Ekwebene et al., 2021** **[44]**	Nigeria	**Study design:** A quantitative study **Population target:** General adult population (aged ≥ 18 years) **Sample size:** 445	53.5%				
**Elhadi et al., 2021** **[28]**	Libya December 2020	**Study design:** A quantitative cross-sectional study **Population target:** General adult population (aged ≥ 18 years) (including medical students and HCW) Sample size: GP-11120	60.6%				
**Eniade et al., 2021** **[45]**	Nigeria December 2020	**Study design:** A quantitative cross-sectional study **Population target:** General adult population (aged ≥ 18 years) **Sample size:** 368	40.5%				
**Gunawardhana et al., 2022** **[29]**	Cameroon 1 June 2021 to 14 July 2021	**Study design:** A quantitative cross-sectional study **Population target:** Pregnant women and general population **Sample size:** GP-448	31%				
**Ilesanmi et al., 2021** **[46]**	Nigeria September 2020	**Study design:** A quantitative cross-sectional study **Population target:** General population **Sample size:** 440					
**Jabessa and Bekele, 2022** **[47]**	Ethiopia August 1st to September 2021	**Study design:** A quantitative cross-sectional study **Population target:** General adult population (aged ≥ 18 years) **Sample size:** 350	29.2%	70.8%			
**James et al., 2021** **[48]**	Nigeria July to August 2022	**Study design:** A quantitative cross-sectional study **Population target:** General adult population (aged ≥ 18 years) **Sample size:** 334	53%				
**Josiah and Kantaris, 2021** **[49]**	Nigeria December 2020	**Study design:** A quantitative cross-sectional study **Population target:** General adult population (aged ≥ 18 years) **Sample size:** 410	48.6%	51.4%			
**Kahn et al., 2022** **[50]**	South Africa 25 August 2021 to 29 October 2021	**Study design:** A quantitative cross-sectional study **Population target:** General adult population (aged ≥ 18 years) **Sample size:** 1662				50.4%	49.6%
**Lamptey et al., 2021** **[51]**	Ghana October to December 2020	**Study design:** A quantitative cross-sectional study **Population target:** General adult population (aged ≥ 18 years) **Sample size:** 1000	54.1%				
**McAbee et al., 2021** **[52]**	Zimbabwe May 2021	**Study design:** A quantitative cross-sectional study **Population target:** General adult population (aged ≥ 18 years) **Sample size:** 551	55.7%	25.6%		18.7%	
**Mesesle, 2021a** **[53]**	Ethiopia March to April 2021	**Study design:** A quantitative cross-sectional study **Population target:** General adult population (aged ≥ 18 years) **Sample size:** 425					
**Mesesle, 2021b** **[54]**	Ethiopia April 2021	**Study design:** A quantitative cross-sectional study **Population target:** General adult population (aged ≥ 18 years) **Sample size:** 415	45.5%	54.5%			
**Mohamud et al., 2021** **[55]**	Somalia October to December 2020	**Study design:** A quantitative cross-sectional study **Population target:** General population **Sample size:** 500	36.8%	63.2%			
**Natuhoyila et al., 2021** **[56]**	The Democratic Republic of Congo January to March 2021	**Study design:** A quantitative cross-sectional study **Population target:** General adult population (aged ≥ 18 years) **Sample size:** 11,971					
**Ocholla et al., 2021** **[57]**	Kenya March 2021	**Study design:** A quantitative cross-sectional study **Population target:** General population **Sample size:** 171	52.4%				
**Okai and Abekah-Nkrumah, 2022** **[58]**	Ghana 18 May 2021 to 14 July 2021	**Study design:** A quantitative cross-sectional study **Population target:** General adult population (aged ≥ 18 years) **Sample size:** 362	62.7%	37.7%			
**Omar and Hani, 2021** **[59]**	Egypt January to March 2021	**Study design:** A quantitative cross-sectional study **Population target:** General adult population (aged ≥ 18 years) **Sample size:** 1011	25%	21%	54%		
**Orangi et al., 2021** **[60]**	Kenya February 2021	**Study design:** A quantitative cross-sectional study **Population target:** General adult population (aged ≥ 18 years) **Sample size:** 4136					
**Shah et al., 2022** **[61]**	Kenya November 2021 to January 2022	**Study design:** A quantitative cross-sectional study **Population target:** General adult population (aged ≥ 18 years) **Sample size:** 3996				68.8%	
**Tlale et al., 2022** **[62]**	Botswana 1 February 2021 to 28 February 2021	**Study design:** A quantitative cross-sectional study **Population target:** General adult population (aged ≥ 18 years) **Sample size:** 5300	73.4%				
**Tobin et al., 2021** **[63]**	Nigeria July 2020 to August 2020	**Study design:** A quantitative cross-sectional study **Population target:** General adult population (aged ≥ 18 years) **Sample size:** 1228	50.2%				
**Toure et al., 2022** **[30]**	Guinea 23 March 2021 to 25 August 2021	**Study design:** A mixed method cross-sectional study **Population target:** General adult population (aged ≥ 18 years) and HCW **Sample size:** GP–3663				31%	
**Wafula et al., 2022** **[64]**	Uganda March 2021	**Study design:** A quantitative cross-sectional study **Population target:** General adult population (aged ≥ 18 years) **Sample size:** 1053	57.8%	16%	26.2%		

**Table 3:** Predictors of COVID-19 vaccine uptake among the general population in Africa.

Country and Study ID	Attitudes and Perceptions towards COVID-19 Vaccines	Reasons for Acceptance/ Non-Acceptance	Determinants Affecting the Vaccine-Related Outcome (Significantly Associated)	Information Sources for COVID-19 Vaccines
**Ethiopia** **[31]**	Negative attitude		**Acceptance:** Age (≥46 years) Education level (secondary and above) Presence of comorbidities Having a good knowledge of COVID-19	Interpersonal relationships Mass media Social media Internet
**Ghana** **[32]**	Hesitant attitude	**Acceptance:** Desire to protect people Confidence in the COVID-19 vaccines A public health responsibility Awareness of possible side effects **Non-acceptance:** Safety concerns Perceived lack of information Effectiveness concerns Vaccine inaccessibility	**Acceptance:** Gender (female) Age (older)	Mass media Social media Interpersonal relationships Other
**Nigeria** **[33]**		**Non-acceptance:** Perceived scientific uncertainty Belief in one’s immune system Safety concerns	**Acceptance:** Age Geographical region **Non-acceptance:** Age Education level	
**Nigeria** **[34]**	Positive perception	**Non-acceptance:** Safety concerns	Acceptance: Education level (diploma and above) Prior diagnosis of COVID	Mass media Social media HCWs Interpersonal relationships Other
**Ghana** **[35]**		**Acceptance:** Desire to protect people Increased education about COVID-19 vaccines Observing others get the COVID-19 vaccine Vaccines are available free of charge Receiving a vaccination certificate **Non-acceptance:** Vaccine inaccessibility Safety concerns Subscribing to misinformation or conspiracies		
**17 African countries** **[26]**	Negative perception	**Acceptance:** Prior diagnosis of COVID Having a positive perception towards the COVID-19 vaccine **Non-acceptance:** Subscribing to misinformation or conspiracies		Social media Mass media Interpersonal relationships Religious or traditional leaders Other
**Somalia** **[36]**		**Non-acceptance:** Effectiveness concerns Safety concerns Belief in one’s immune system Doubts about the seriousness of the pandemic	**Non-acceptance:** Gender (female)	
**Multiple** **African** **countries** **[27]**		**Non-acceptance:** Safety concerns Preferred alternative treatment to the COVID-19 vaccine	**Non-acceptance:** Age (older) Gender (female) Employment status Income level Residence (urban area) Negative experience with vaccines	HCWs Mass media Government Religious or traditional leaders Other
**Ethiopia** **[37]**	Hesitant attitude	**Non-acceptance:** Safety concerns	**Acceptance:** Gender (female) Age (<30) Marital status Residence Occupation Religion (Muslim) Education level (tertiary)	
**Multiple** **African** **countries** **[25]**				
**South Africa** **[38]**	Opposing attitudes	**Acceptance:** Desire to protect people To return to normality **Non-acceptance:** Lack of trust in stakeholders		
**Nigeria** **[39]**	Hesitant attitude	**Acceptance:** Confidence in COVID-19 vaccines **Non-acceptance** Subscribing to misinformation or conspiracies		
**Nigeria** **[40]**		**Non-acceptance:** Safety concerns Effectiveness concerns Negative experience with vaccines	**Non-acceptance:** Safety concerns Perceived scientific uncertainty Effectiveness concerns Negative experience with vaccines	
**The** **Democratic Republic of Congo** **[41]**		**Non-acceptance:** Subscribing to misinformation or conspiracies Safety concerns Effectiveness concerns	**Acceptance:** Income level (middle-and high-level) Prior diagnosis of COVID	Interpersonal relationships Mass media Religious or traditional leaders Social media
**Mozambique** **[42]**		**Acceptance:** Confidence in COVID-19 Vaccines **Non-acceptance:** Effectiveness concerns Safety concerns Subscribing to misinformation or conspiracies		
**Uganda** **[43]**		**Non-acceptance:** Safety concerns	**Acceptance** Age (18–20 years) Education level (primary) Occupation Religion (Christian) Marital status (married) Residence (rural area) Income level	
**Nigeria** **[44]**	Hesitant attitude	**Non-acceptance:** Safety concerns Religious beliefs **Acceptance:** Confidence in COVID-19 vaccines		
**Libya** **[28]**	Positive attitude	**Acceptance:** Confidence in the COVID-19 vaccines **Non-acceptance:** Subscribing to misinformation or conspiracies	**Acceptance:** Age (31–50 years) Marital status (married) Geographical region Prior diagnosis of COVID Knowing someone who tested positive for COVID Efficacy of 70% and above **Non-acceptance:** Loss of someone to COVID-19	Mass media Social media Internet
**Nigeria** **[45]**		**Non-acceptance:** Lack of trust in stakeholders Safety concerns	**Acceptance:** High perceived susceptibility to contracting COVID Age (≥40)	Social media Mass media Interpersonal relationships
**Cameroon** **[29]**	Hesitant attitude	**Non-acceptance:** Safety concerns Effectiveness concerns Perceived lack of information Subscribing to misinformation or conspiracies **Acceptance:** Receiving COVID-19 vaccines from an African country	**Acceptance:** Being a parent Education level (secondary)	HCWs Social media Mass media Interpersonal relationships Government Religious or traditional leaders Other
**Nigeria** **[46]**	Positive perception			Mass media Social media
**Ethiopia** **[47]**			**Acceptance:** Age (≥50) Income level (low) Low perception level towards COVID-19 vaccines Employment status (unemployed) low level of acceptance of COVID-19 vaccines Unwilling to test for COVID Education level (low)	
**Nigeria** **[48]**	Positive attitude and negative perception	**Non-acceptance:** Safety concerns	**Acceptance:** Age (older) Gender Education level Marital status	
**Nigeria** **[49]**		**Non-acceptance:** Doubts about the seriousness of the pandemic Receiving COVID-19 vaccines from a Western/European country Perceived scientific uncertainty **Acceptance:** Receiving COVID-19 vaccines from an African country Receiving COVID-19 vaccines from a Muslim country	**Acceptance:** Gender Education level Religious beliefs Employment status Income level High perceived susceptibility to contracting COVID Trust in stakeholders	
**South Africa** **[50]**		**Non-acceptance:** Perceived lack of information Safety concerns Vaccine inaccessibility	**Acceptance:** Age (older) Confidence in the COVID-19 vaccines	
**Ghana** **[51]**		**Acceptance:** Age Marital status Education level Occupation		
**Zimbabwe** **[52]**			**Acceptance:** Practising COVID-19 prevention measures Confidence in the COVID-19 vaccines The COVID-19 vaccines are being recommended by the Ministry of Health and World Health Organisation Vaccines are available free of charge Vaccine accessibility Education level (secondary and tertiary) Gender (male)	
**Ethiopia** **[53]**	Positive attitude and negative perception			Mass media
**Ethiopia** **[54]**		**Non-acceptance:** Perceived lack of information Safety concerns **Acceptance:** Access to the media	**Acceptance:** Gender Education level Use of mass media Received childhood vaccines Knowing someone who tested positive for COVID	Mass media
**Somalia** **[55]**	Negative attitude	**Non-acceptance:** Safety concerns Effectiveness concerns Perceived scientific uncertainty		
**The** **Democratic** **Republic of Congo** **[56]**	Negative perception	**Non-acceptance:** Effectiveness concerns Safety concerns Subscribing to misinformation or conspiracies		
**Kenya** **[57]**		**Non-acceptance:** Safety concerns	**Acceptance:** Occupation	Mass media Social media HCWs Interpersonal relationships
**Ghana** **[58]**		**Non-acceptance:** Safety concerns Perceived scientific uncertainty Low perceived susceptibility to contracting COVID		
**Egypt** **[59]**	Negative attitude	**Non-acceptance:** Safety concerns	**Non-acceptance:** Gender (female) Residence (urban area) Education level (tertiary) Marital status (married) Rejected flu vaccines in the past Lack of trust in stakeholders	
**Kenya** **[60]**	Hesitant attitude	**Acceptance:** High perceived susceptibility to contracting COVID **Non-acceptance:** Safety concerns Effectiveness concerns Religious beliefs	**Non-acceptance:** Residence (rural area) Safety concerns Effectiveness concerns Vaccine inaccessibility Religious beliefs	
**Kenya** **[61]**		**Non-acceptance:** Safety concerns		
**Botswana** **[62]**		**Non-acceptance:** Perceived scientific uncertainty Safety concerns	**Acceptance:** Gender (male) Age (55–64 years) Education level (primary) Willingness to wear a mask Employment status Presence of comorbidities	Mass media Social media Internet Government
**Nigeria** **[63]**		**Acceptance:** Self-protection Desire to protect people COVID-19 vaccines are being recommended by HCWs Presence of chronic illness **Non-acceptance:** Safety concerns Effectiveness concerns Doubts about the seriousness of the pandemic Afraid of needles	**Acceptance:** Age (≥24 years) Religion (Muslim) Gender (male) Trust in stakeholders Willing to pay and travel for the COVID-19 vaccine Vaccinating during an outbreak	Social media Internet Mass media HCWs Interpersonal relationships
**Guinea** **[30]**	Negative perception	**Non-acceptance:** Having a negative perception towards the COVID-19 vaccine Lack of trust in stakeholders Safety concerns	**Acceptance:** Marital status (single) Education level Non-pregnant women Confidence in the COVID-19 vaccines Age Vaccine eligibility **Non-acceptance:** Higher vaccine knowledge Having a negative attitude toward the COVID-19 vaccine	Mass media Social media Interpersonal relationships Government
**Uganda** **[64]**		**Non-acceptance:** Safety concerns Effectiveness concerns Perceived lack of information Low perceived susceptibility to contracting COVID Prior diagnosis of COVID Belief in one’s immune system	**Acceptance:** Geographical region Age (55–64 years) High perceived susceptibility to contracting COVID Developing severe disease and side effects	Mass media

## Data Availability

Not applicable.

## Abbreviations

CDC	Centers for Disease Control and Prevention
CHW	Community Healthcare Worker
COVID-19	Coronavirus disease 2019
DRC	Democratic Republic of the Congo
GP	General Population
HCW	Healthcare Worker
HIV/AIDS	Human immunodeficiency virus/acquired immunodeficiency syndrome
HPP	Health Promotion Practitioner
IDVI	Infectious Disease Vulnerability Index
MERS-Cov	Middle East respiratory syndrome coronavirus
PRISMA-ScR	Preferred Reporting Items for Systematic reviews and Meta-Analyses extension for Scoping Reviews
SARS-CoV	Severe acute respiratory syndrome
SARS-CoV-2	Severe acute respiratory syndrome coronavirus 2
TB	Tuberculosis
VH	Vaccine Hesitancy
VPD	Vaccine-preventable diseases
WHO AFRO	World Health Organization African Region
WHO	World Health Organization

## Appendix A. Search Strategy

**Table A1:** EBSCO host search strategy.

Search ID#	Search Terms	Search Options	Actions
S5	(Attitude and perception towards the COVID-19 vaccine) AND SU Africa OR TI (vaccine hesitancy or vaccine refusal or vaccine acceptance or vaccine uptake)	Limiters—Full Text; Published Date: 20200101–20211231; Hidden NetLibrary Holdings Expanders—Apply equivalent subjects Search modes—Boolean/Phrase	(247)
